# Double lag-screw compression for optimal fixation of intertrochanteric fractures with large fragment gap: A technical note

**DOI:** 10.1051/sicotj/2023005

**Published:** 2023-04-19

**Authors:** Panagiotis Karampinas, Athanasios Galanis, Eftychios Papagrigorakis, Michail Vavourakis, Anastasia Krexi, Spiros Pneumaticos, John Vlamis

**Affiliations:** Department of Orthopaedic Surgery, National & Kapodistrian University of Athens, KAT General Hospital Athens Greece

**Keywords:** Double compression, Lag screw, Peritrochanteric fracture, Cephalomedullary nail, Screw cut-out, Fracture gap

## Abstract

Cephalomedullary nailing of unstable intertrochanteric fractures has been established as a fruitful surgical approach with relatively limited complications. Anatomic fracture reduction and proper implant positioning are vital to attaining a favorable long-term surgical outcome. Appropriate intraoperative fracture compression augments stability and invigorates healing. The amount of compression permitted by cephalomedullary nails cannot always adequately reduce large fragment gaps. This paper presents a novel technical trick of double compression of the fracture site, in order to achieve the essential extra compression and reduction when required, thus decreasing the risk of postoperative implant cut-out. The technique was used in 14 out of 277 peritrochanteric fractures treated with cephalomedullary nailing in our trauma center for 12 months, with satisfactory outcomes regarding both fracture site union and postoperative functional capacity.

## Introduction

Peritrochanteric fracture treatment aims to attain a stable fracture fixation for the patients to return to their pre-fracture activities as rapidly as possible. Over the past decades, outcomes have confoundedly improved with operative treatment [1]. However, even nowadays, this injury’s complication and mortality rates remain moderately high. The most frequently reported complication of using a cephalomedullary nailing system is implant cut-out, in which the lag screw falls into displacement through the femoral head. Many factors, such as age, bone quality, fracture pattern, implant design, lag screw position, and reduction level, can contribute to an increased risk of screw cut-out [2].

It is paramount to practice diligent operative techniques to achieve anatomic fracture reduction because proper implant placement cannot be accomplished prior to an anatomic reduction [3]. TAD and CalTAD values are of great significance concerning the risk for postoperative cut-out [4, 5]. Precise intraoperative fracture compression enhances stability and promotes fracture healing, resulting in greater benefits for the patient with early mobilization and full weight-bearing while avoiding the complications of prolonged bed rest [2–4]. Intraoperative positive anteromedial cortical support reduction is fundamental to eradicate the risk of various postoperative mechanical complications, regardless of the type of implant employed [6–10].

**Figure 1:**
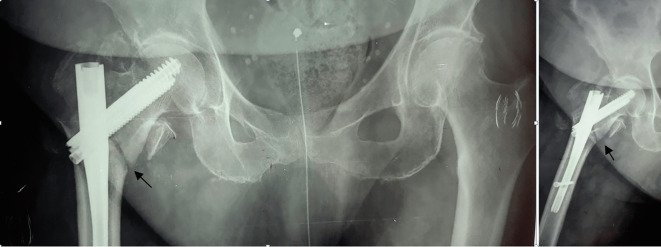
Final X-rays of unstable peritrochanteric fracture treated with cephalomedullary nailing (Trigen InterTAN, Smith & Nephew) and intraoperative compression at the maximum amount permitted.

**Figure 2:**
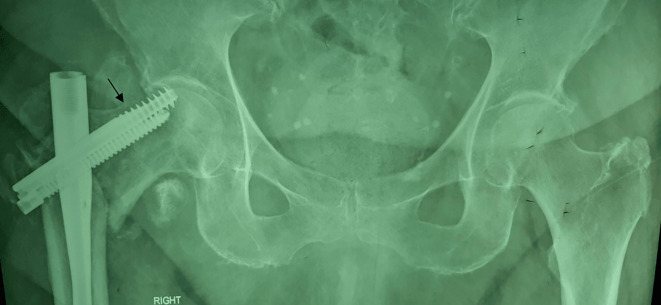
Lag screw cut-out 3 months postoperatively.

In many cases, a large fracture gap (greater than 10 mm) cannot be satisfactorily reduced with the amount of compression permitted by dual lag-screw cephalomedullary nailing systems. When the fracture gap cannot be eliminated, and complete compression of the fracture cannot be achieved, the risk of lag screw cut-out and osteosynthesis failure is high (Figures 1 and 2).

To avoid these complications, we recommend a technical trick of double compression (two-stage compression) of the fracture site to attain gradually more than 13 mm of compression and always in control by the surgeon. When utilizing a dual lag-screw cephalomedullary nail for fixation of intertrochanteric fractures with large fracture gaps, complete compression of the fracture site and absolute medial cortex reduction can be safely achieved with our trick. This offers an utterly stable osteosynthesis and facilitates the postoperative management of challenging unstable peritrochanteric fractures, allowing immediate full weight-bearing, while this is not feasible with the conventional techniques as sufficient medial wall compression cannot be accomplished.

To our knowledge, there is no other surgical technique described in the existing literature that resolves the problem of remaining fracture gap after implementing the full compression offered by the cephalomedullary nailing systems.

## Materials and methods

In our trauma center, 277 patients sustaining an intertrochanteric fracture were admitted and operated on using a dual lag-screw cephalomedullary nailing system (Trigen InterTAN, Smith & Nephew, UK) during a 12-month length of time. During the operation, a fracture gap greater than 13 mm was present in 14 cases, in which the undermentioned double compression technique was applied. The technique is not included in the company’s manual for Trigen InterTAN, Smith & Nephew, UK (10 + 3 mm maximum compression is depicted).

**Figure 3:**
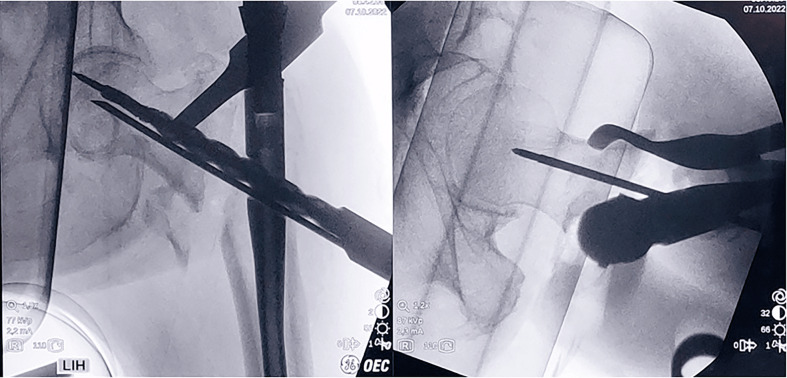
Position the guide in the correct TAD position and calculate the length of the interlocking lag screw. If we measure 100 mm, we deduct 15 mm which is the fracture gap we need to reduce, thus the length of the lag screw should be 85 mm. The compression screw length is 80 mm.

The technique is described as follows:

After executing the anatomical reduction of the fracture and a large fracture gap is observed, we proceed with the standardized steps of nail insertion. We confirm the guide pin position in the anteroposterior (AP) and lateral planes. The guide pin should be at the femoral head’s center in both views, with a tip-apex distance (TAD) of less than 25 mm (Figure 3). At this point, we calculate the lag screw length, which should equal the drilled depth length minus the desired amount of compression (e.g. drilling depth 100 mm, compression 10 + 5 mm, lag screw length 85 mm).

Proceed with the standardized step of drilling for interlocking compression-screw insertion and then insert the antirotation blade (as it is a dual-head screw implant). Carry on with the established procedure for lag screw drilling and insertion. Under fluoroscopy imaging, advance the lag screw manually to the 0 mm mark on the lag screwdriver and then to the 10 mm mark. The mark on the lag screwdriver represents the system’s available amount (10 mm maximum) of compression, which is the gap reduction length offered by the system controlling the procedure.

Remove the anti-rotation bar and attach the interlocking compression screw packaged with the lag screw. Advance the compression screw, and after releasing traction, compression is achieved by advancing the compression screw assembly until the 0 mm mark on the lag screwdriver is visible (first compression).

**Figure 4:**
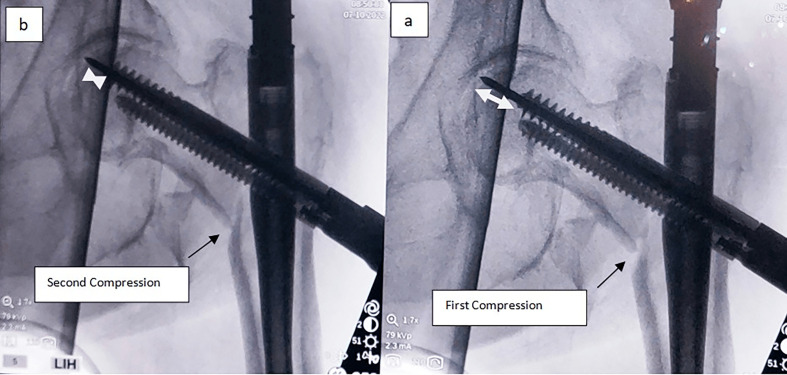
Sufficient space under the subchondral region is evident (a), which offers us the ability to advance further the lag screw (b), in order to proceed to the second compression and maximum reduction of the gap (arrow).

It is recommended to halt the compression when the 0 mm mark appears. However, extra compression (2–3 mm) may be accomplished by advancing the compression screw until the red mark on the lag screwdriver is discernible (in the Company’s manual note: it is not recommended to exceed 10 mm of compression). Until this point, the common surgical procedure is performed, and at the c-arm’s monitor, we descry a remaining fracture gap (5 mm). If calculated correctly, there is space between the subchondral area of the femoral head and the lag screw to advance (5 mm) (Figure 4). The lag screw can be advanced for an extra 5 mm.

At this point, we proceed with the steps for the second compression. Remove the compression screw and reinsert the anti-rotation bar in its position. Under constant fluoroscopy-imaging assistance, advance the lag screw with the proper screwdriver at the desired depth (advanced 5 mm and in the subchondral position of 5 mm). Do not advance the lag screw without the anti-rotation blade in place.

At the lag-screw’s screwdriver, notice the mark of the extra depth advanced by the lag screw (5 mm). Remove the anti-rotation bar and reinstall the compression screw. Then, commence the second compression of the interlocking lag screw, the extent of which is determined by the amount of resistance received from the fracture gap compression and the fluoroscopic imaging.

**Figure 5:**
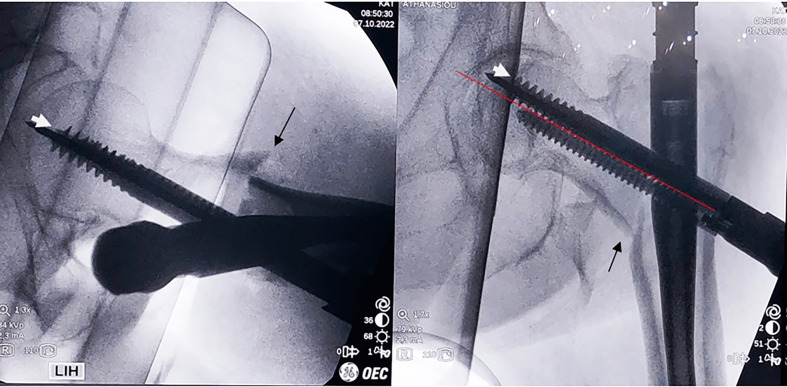
Final images depicting the ultimate reduction of the fracture gap at the maximum (15 mm) desirable measure, with optimal TAD and a double compression technique.

Keep on with fracture site compression until the optimal outcome is evident in both views (Figure 5).

Second fracture compression is acquired. Proceed with the standardized steps for locking the pre-loaded set screw and the nail distally. Medial wall compression and lag screw locking provide stability to the fracture site and the osteosynthesis, promoting fracture healing and immediate full weight-bearing.

## Results

The double compression technique was applied to all 14 cases of peritrochanteric fracture with a large gap (greater than 13 mm) with a 100% success rate. No intraoperative and postoperative complications were observed. The technique was completely controlled by the surgeon during the operation. It was effortlessly repeated in all cases, without the learning curve arduousness. There was a positive reduction and compression of the fracture site in all cases. We did not detect any lag-screw cut-out during the first year of follow-up. Full weight-bearing was achieved immediately postoperatively in all cases. At the 1-year follow-up, a fracture union was observed in the hip X-rays of all 14 patients, which were pain-free VAS 0.6 out of 10 and presented with a near-perfect postoperative Harris Hip Score [92.3 out of 100, range (84–97)].

## Discussion

Particular attention should be paid to fracture site reduction and lag screw positioning to diminish mechanical complications such as lag screw cut-out, which aggravate morbidity and mortality associated with proximal femur fractures [4]. Studies have provided the first clinical evidence bolstering the validity and reliability of CalTAD as a predictor of lag screw cut-out, which is a measurement method that favors the inferior–central region of the femoral head for placing the lag screw [4, 5]. The value of CalTAD appears to be more effectual than the TAD value in predicting the risk of the cut-out in the postoperative period. However, the discrepancy between the two methods is minimal [5]. In addition, searching the existing literature, no studies demonstrate greater sensitivity and specificity of CalTAD than TAD and vice-versa. It seems preferable to utilize a nailing system with a double cephalic screw and static distal locking. In case of poor fracture reduction and/or unstable fixation, constrained weight-bearing alone is insufficient to prevent the occurrence of cut-out [5]. The cephalomedullary nail type is not a risk factor for implant cut-out. Suboptimal lag screw positioning and imperfect reduction are correlated with an enhanced risk of screw cut-out [6]. Risk factors of single-lag screw implant failure also apply to dual-lag screw implants. TAD and CalTAD are significant factors regarding the incidence of the cut-out in dual-lag screw implants. Screw cut-out can be minimized by optimizing lag screw placement [7]. The incidence of cut-out can be confined when performing meticulous reduction and stable fixation by avoiding TAD > 34.8 mm and CalTAD > 35.2 mm [5].

During fracture reduction, cortical support should be ensured for both AP and lateral views. It is essential to be vigilant regarding the positive cortical support in the lateral view [8]. Superior clinical outcomes and lower complication rates are achieved by efficiently reducing the medial and anteromedial cortices. The sustainable stability and anti-rotational function provided by satisfactory fracture reduction play a crucial role in maintaining the fragment positions in place and reducing the incidence of complications [9]. Also, fracture reduction with valgus alignment allows restricted sliding of the head–neck fragment to contact the femoral shaft. As a result, secondary stability is accomplished, providing an advantageous mechanical environment for fracture healing [8, 9]. A negative lateral position of the anterior cortex may be highly predictive of the final loss of the anteromedial cortical buttress [10]. Besides the AP and lateral projections, an anteromedial oblique view of 30° is regarded as a very useful means to assess the quality of fracture reduction in terms of anteromedial cortical apposition [11]. In our intraoperative clinical practice, we carry out the compression of the fracture, single or double, in both lateral and oblique fluoroscopic views, controlling the medial and anteromedial cortical positions.

Poor reduction and larger calcar fracture gapping in the AP and lateral views are risk factors leading to alterations in anteromedial cortical support [10]. Mechanical complications transpire more frequently in the presence of lost anteromedial cortex support. Calcar fracture gapping in the AP and lateral views should be restrained to be less than 4 mm. Compression of the fragments is beneficial, as it contracts the sliding of the head and neck fragment and secures anteromedial cortical support at the anteromedial corner [12]. Femoral offset and precision of neck-shaft angle restoration were correlated with TAD outcomes. During the closed reduction procedure, rigorous intraoperative control of femoral offset and neck-shaft angle distinguishes non-optimal reductions [13]. With our technical note of double compression in two-screw cephalomedullary nailing systems, we eliminate any fracture gap remaining by implant limitations and reach a stable osteosynthesis of the peritrochanteric fracture.

Meta-analysis data suggest that integrated twin compression screw cephalomedullary nails are clinically more efficacious compared to single screw derotation cephalomedullary nails, resulting in fewer postoperative complications, revisions, and reduced pain scores [14]. On the other hand, no difference was observed in terms of Harris Hip Scores and non-unions, while single screw anti-rotation nails are correlated to significantly reduced blood loss and fluoroscopy use [14]. It is deduced that despite the several complications reported following the treatment of peritrochanteric fractures, double-screw implants provide sufficient reduction and intraoperative compression of the fracture, promoting healing in a stable environment, with good or excellent functional outcomes in the majority of the cases [15]. However, even these implants, are associated with reduced Harris Hip Scores when the patients are aged over 85, their pre-fracture ambulation is considerably limited and the fracture types are AO/OTA-31-A3 [15]. It is controversial whether integrated dual lag screws provide superior functional outcomes compared to single lag screw cephalomedullary nails, as present-day data are inadequate. [16] Nonetheless, meta-analysis evidence corroborates the estimation that dual-head screw implants are related to fewer revisions and complications such as screw migration and varus collapse [16].

At this point, we add an extra benefit in the use of integrated twin compression screw cephalomedullary nails, which is the capability of a second (double) compression of the fracture site intraoperatively. This offers unstable fractures with a large gap, and a proper and stable healing environment. It is an undemanding and repeatable procedure.

## Conclusion

Absolute reduction of the fracture gap and intraoperative compression of the fracture site is requisite to attain stable and safe osteosynthesis. Immediate weight-bearing of the fracture can be achieved, avoiding complications such as lag screw cut-out. The technical note of double compression presented by the authors is a safe and repeatable procedure. It can provide the crucial extra reduction and compression of the fracture gap when necessary, constantly controlled by the surgeon, exceeding the features of integrated dual lag-screw cephalomedullary nails.
